# Metabolome Response to Glucose in the β-Cell Line INS-1 832/13*[Fn FN2]

**DOI:** 10.1074/jbc.M112.414961

**Published:** 2013-02-20

**Authors:** Matthew A. Lorenz, Mahmoud A. El Azzouny, Robert T. Kennedy, Charles F. Burant

**Affiliations:** From the Departments of ‡ Chemistry,; § Pharmacology, and; ¶ Internal Medicine, University of Michigan, Ann Arbor, Michigan 48105

**Keywords:** Beta Cell, Insulin Secretion, Intermediary Metabolism, Metabolism, Metabolomics

## Abstract

Glucose-stimulated insulin secretion (GSIS) from pancreatic β-cells is triggered by metabolism of the sugar to increase ATP/ADP ratio that blocks the K_ATP_ channel leading to membrane depolarization and insulin exocytosis. Other metabolic pathways believed to augment insulin secretion have yet to be fully elucidated. To study metabolic changes during GSIS, liquid chromatography with mass spectrometry was used to determine levels of 87 metabolites temporally following a change in glucose from 3 to 10 mm glucose and in response to increasing concentrations of glucose in the INS-1 832/13 β-cell line. U-[^13^C]Glucose was used to probe flux in specific metabolic pathways. Results include a rapid increase in ATP/ADP, anaplerotic tricarboxylic acid cycle flux, and increases in the malonyl CoA pathway, support prevailing theories of GSIS. Novel findings include that aspartate used for anaplerosis does not derive from the glucose fuel added to stimulate insulin secretion, glucose flux into glycerol-3-phosphate, and esterification of long chain CoAs resulting in rapid consumption of long chain CoAs and *de novo* generation of phosphatidic acid and diacylglycerol. Further, novel metabolites with potential roles in GSIS such as 5-aminoimidazole-4-carboxamide ribotide (ZMP), GDP-mannose, and farnesyl pyrophosphate were found to be rapidly altered following glucose exposure.

## Teaser

**Background:** The biochemical pathways underlying glucose-stimulated insulin secretion have not been fully elucidated.

**Results:** Mass spectrometry analysis revealed rapid and substantial metabolic reprogramming evoked by glucose in INS-1 cells.

**Conclusion:** Metabolomics allowed testing and generation of multiple hypotheses regarding glucose effects in insulin-secreting cells.

**Significance:** Insights into the biochemical basis of glucose-stimulated insulin secretion are critical for understanding root causes of type 2 diabetes.

## Introduction

β-Cells in islets of Langerhans secrete insulin in response to increased blood glucose. An acute increase in glucose evokes a rapid release of insulin, which is sustained for a short period, designated as 1st phase, followed by an extended period of lower secretion (2nd phase). The metabolic pathways that facilitate 1st and 2nd phase of glucose-stimulated insulin secretion (GSIS)^3^ by β-cells are not fully understood (1). GSIS is dependent upon glucose metabolism and is thought to be triggered by closure of K_ATP_ channels secondary to an increase in the ATP/ADP ratio. Closure of K_ATP_ channels causes membrane depolarization, opening of voltage-sensitive Ca^2+^ channels, and subsequent exocytosis of a releasable pool of insulin vesicles (2). Evidence supports the concept that other metabolic processes also facilitate GSIS in K_ATP_-independent or amplifying pathways (3–5). A variety of metabolic coupling factors (*e.g.* NADPH, long-chain acyl-CoAs, and glutamate) and pathways (*e.g.* pyruvate/citrate, pyruvate/isocitrate, pyruvate/malate, and glycerolipid/fatty acid cycling) have been implicated in both triggering and amplifying GSIS (3–5).

Measurement of metabolite changes in β-cells that correlate with GSIS has been limited to measuring a relatively small set of metabolites at extended time periods that cannot distinguish important alterations that occur in earliest phases of insulin secretion (6). In this work, we employed a recently developed liquid chromatography time of flight-mass spectrometry (LC-TOF-MS) method to measure signal for hundreds of metabolites in INS-1 832/13 cells following exposure to glucose (7). We determined the identity and levels of 87 of these metabolites in response to different glucose concentrations and at short intervals after exposure to an increase in glucose. Specific compounds monitored included metabolites associated with glycolysis, the pentose phosphate shunt, and the tricarboxylic acid (TCA) cycle as well as an array of nucleotides and fatty acid species. We also utilized isotopologue analysis of metabolites following U-[^13^C]glucose stimulation to assess flux through specific pathways. The results allowed us to confirm or modify prevailing hypotheses regarding metabolism and its relationship to insulin secretion and identify new pathways that may play a role in the dynamics of insulin secretion following glucose exposure.

## EXPERIMENTAL PROCEDURES

### 

#### 

##### Materials

INS-1 832/13 (8) cells were kindly provided by Dr. Christopher Newgard (Sarah W. Stedman Nutrition and Metabolism Center, Duke University, Durham, NC). All chemicals were purchased form Sigma-Aldrich unless otherwise noted. HPLC grade acetonitrile was purchased from Burdick & Jackson (Muskegon, MI). RPMI media, fetal bovine serum, 4-(2-hydroxyethyl)-1-piperazineethanesulfonic acid (HEPES), and penicillin-streptomycin were purchased from Invitrogen (Carlsbad, CA).

##### Cell Culture

INS-1 832/13 cells cultured in RPMI supplemented with 2 mm glutamine, 1 mm sodium pyruvate, 10% FBS, 10 mm HEPES, 100 units/ml penicillin, 100 μg/ml streptomycin, 250 ng/ml amphotericin B, and 50 μm β-mercaptoethanol. Cells were plated at a density of ∼14 × 10^3^ cells/cm^2^ and grown in either 6- or 10-cm culture dishes at 37 °C and 5% CO_2_ in a humidified atmosphere to ∼70% confluence over ∼5 days prior to experimentation. Cells were preincubated in supplemented RPMI containing 3 mm glucose for ∼20 h prior to experimentation. Krebs-Ringer-HEPES buffer (KRHB) was prepared containing 10 mm glucose, 20 mm HEPES, 118 mm NaCl, 5.4 mm KCl, 2.4 mm CaCl_2_, 1.2 mm MgSO_4_, and 1.2 mm KH_2_PO_4_ adjusted to pH 7.4 with HCl.

##### Glucose Stimulation Dose Response

Following preincubation, culture media was replaced with KRHB containing 0, 2, 5, 10, or 20 mm glucose + 0.2% BSA. Cells were incubated for 30 min after which an aliquot of buffer was removed for insulin measurement. Metabolism was immediately quenched and metabolites extracted as described previously (7).

##### Glucose Stimulation Time Course and Flux

Cells were transferred to KRHB containing 0.5 mm glucose and 0.2% BSA for 30 min prior to stimulation. Glucose was increased to 10 mm glucose by adding an aliquot of 1 m glucose stock. The media was sampled for insulin measurement 2–90 min after initial transfer to 0.5 mm KRHB (both pre- and post- stimulation). For metabolite measurements, cells were treated as indicated above (without addition of BSA) and cell plates quenched from 25 to 75 min after transfer to KRHB (5 min before to 45 min after increasing glucose to 10 mm). Carbon flux through glucose was also assessed by stimulating cells with U-[^13^C]glucose using the same protocol. In some studies, cells were pretreated with 10 mm phenylsuccinate (Sigma-Aldrich) for 10 min to inhibit the mitochondrial oxoglutarate carrier (9).

For insulin and metabolite measurements with 5-amino-1-β-d-ribofuranosyl-imidazole-4-carboxamide riboside (AICAR) treatment, stimulation was conducted by conditioning cells in KRHB containing 0.5 mm glucose for 30 min and replacing the buffer with KRHB containing 10 mm glucose with or without 25 μm AICAR. Incubation buffer was sampled for insulin and plates quenched from 10 to 60 min following stimulation.

##### Insulin Measurement

Aliquots of KRHB were briefly stored on ice, centrifuged at 3000 rpm for 3 min to pellet any suspended cells, and an aliquot of supernatant was transferred to a fresh vial. Samples were stored at −20 °C and assayed using Rat/Mouse insulin ELISA Kit (Millipore, Billerica, MA). Insulin secretion rate was calculated by dividing the difference in insulin concentration of 2 consecutive time points by the time elapsed between sampling.

##### Metabolite Measurement

Cell plates were rinsed, metabolism quenched, and metabolites extracted using the procedure described previously (7). Briefly, cell plates were rapidly rinsed with water and quenched with liquid nitrogen. Metabolites were extracted with 75% 9:1 methanol:chloroform/25% water and assayed by high performance liquid chromatography with time-of-flight mass spectrometry (HPLC-TOF-MS). Chromatographic separations were performed with an Agilent Technologies (Santa Clara, CA) 1200 HPLC system equipped with a Phenomenex (Torrance, CA) Luna NH_2_ 2.0 × 150 mm, 3 μm HPLC column and a 2.0 × 4 mm guard column using the following conditions: mobile phase A was 100% acetonitrile (ACN); mobile phase B was 100% 5 mm ammonium acetate pH 9.9 with ammonium hydroxide; gradient program was (time, %B, flow rate) 0 min, 20%, 200 μl/min, 20 min, 100%, 200 μl/min, 20.1 min, 100%, 300 μl/min; column temperature was 35 °C; injection volume was 80 μl; autosampler temperature was 4 °C. Lipids were separated on a C18 Capcell column (2 mm bore by 150 mm long packed with 3 μm particles). Mobile phases and gradient were used as described (10). Diacylglycerol and phosphatidic acid detection was performed in positive and negative mode, respectively. Detection was performed on an Agilent Technologies LC/MSD TOF using a dual electrospray ionization (ESI) source in negative-ion mode as described previously (7).

Directed and undirected data processing was performed as described elsewhere (7). Metabolites previously implicated in GSIS (*e.g.* glycolytic and TCA cycle intermediates) were identified using standards, accurate mass, and isotope ratios to confirm peak assignments (see supplemental Table S1). Combined peak areas are used for unresolved isomers (*e.g.* citrate + isocitrate and hexose phosphates). Several metabolites are not reported because of rapid degradation or interconversion. For example, pyruvate and oxaloacetate are not reported because of rapid degradation of oxaloacetate to pyruvate (11). Similarly, glyceraldehyde-3-phosphate and dihydroxyacetone phosphate are unstable in solution (12).

Undirected analysis was performed by determining features (*i.e. m*/*z* signals at a given retention time) that changed in LC-MS peak area following a step change from 0.5 to 10 mm glucose for 25 min. Features were included in the analysis that were detected in every chromatogram and had <40% relative standard deviation (RSD) within each group as determined using Agilent Technologies MassHunter Quantitative software for peak picking and MassProfiler Professional for data alignment and statistical analysis. These metabolites were identified by searching the Human Metabolome Database to match mass, analyzing isotope ratios, and comparing to standards when available (see supplemental Table S1).

For relative quantification of identified metabolites, peak areas were used. When available, ^13^C-labeled internal standards were added to sample and peak areas measured relative to the internal standard to improve precision (supplemental Table S1). Calibration curves with standards that were available showed linear responses. To ensure that peak areas were linear with concentration even when analyzing a complex matrix of cell extract, standards for 26 analytes were spiked into an extract of INS-1 cells that had been incubated with 0.5 mm glucose for 2 h. Three to five concentration spikes were added to yield a concentration range that spanned that detected in cells. These standard addition experiments showed highly linear response for the majority of metabolites reported (supplemental Fig. S1).

For absolute quantification and determination of metabolite pool size, the standard addition method was used. Cells were stimulated for 30 min with 10 mm glucose and the resulting extract spiked with standards at six different concentrations (see supplemental Table S1 for metabolites quantified in this way). Residual protein was determined by Bradford Assay (13).

For all studies, peak areas were measured from extracted ion chromatograms of [M-H]^−^ metabolite ions with ± 70 ppm detection windows centered on the theoretical mass. [M-2H]^2−^ ions were used for acetyl-CoA (aCoA) and other CoAs to improve sensitivity. Peak areas for internal standards were measured using an identical procedure.

##### Western Blot

Glucose-stimulated cells were placed on ice, washed once with ice cold PBS, and solubilized in 75 μl of Laemmli/extraction buffer (20 mm HEPES pH 7.5, 1% Triton X-100, 20 mm β-glycerophosphate, 150 mm NaCl, 10 mm NaF, 1 mm sodium orthovanadate, and complete protease inhibitor mixture) from Roche Diagnostics (Indianapolis, IN). Anti-acetyl-CoA carboxylase (ACC) and anti-phospho-ACC antibodies were obtained from Cell Signaling and used at 1:1000. Anti-HMG-CoA-reductase was from US Biological and was used at 2 μg/ml. Blots were developed with ECL (Pierce) according to manufacturer's instructions.

##### Statistics

Data are expressed as mean ± 1 S.E. Statistical significance was determined using a non-corrected two-tailed Student's *t* test, unpaired assuming equal variance or ANOVA, as appropriate. A *p* value of < 0.05 was considered significant.

## RESULTS AND DISCUSSION

### 

#### 

##### Insulin Secretion and Static Metabolite Profiles

In these studies we employed LC-TOF-MS to identify and quantify the levels of specific metabolites in INS-1 832/13 cells (1) following glucose exposure and their relationship to insulin release. We used U-[^13^C]glucose in secondary studies to more fully understand the dynamics of metabolite changes.

**FIGURE 1.**
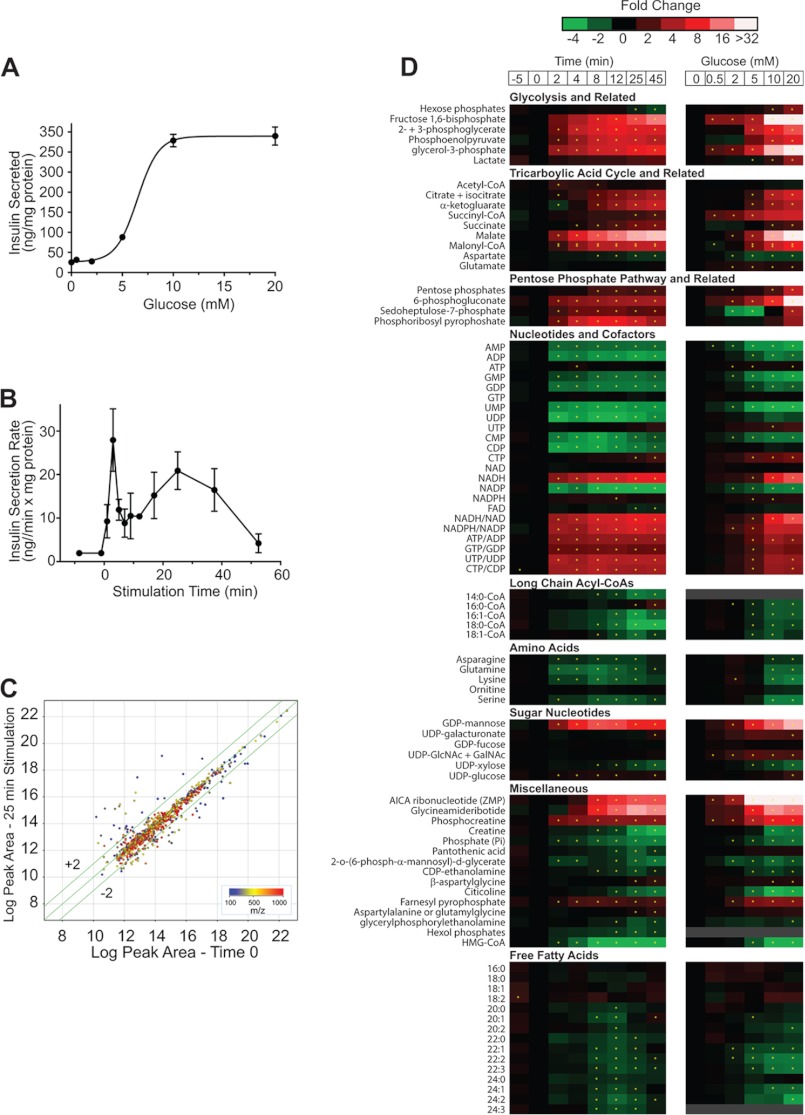
**Temporal and dose-response metabolite profiles and insulin release with glucose stimulation in INS-1 832/13 cells.** *A*, insulin secreted by INS-1 832/13 cells *versus* glucose concentration. Cells incubated in KRBH +0.2% BSA and 0 to 20 mm glucose for 30 min. Error bars are S.E., *n* = 3. *B*, insulin secretion rate *versus* stimulation time. INS-1 cells incubated in KRHB + 0.2% BSA + 0.5 mm glucose for 30 min and stimulated with 10 mm glucose for 50 min. *C*, effect of 10 mm glucose treatment on LC-MS “feature” peak area. The log-log plot is for all features detected at time 0 (0.5 mm glucose) *versus* 25 min of 10 mm glucose stimulation. Features are color coded by *m*/*z*. The 1030 features plotted were detected in all replicates with RSD < 40% for either Time 0 or 25 min groups. 130 feature peak areas change >1.5-fold (shown outside the lines parallel to the correlation line) and are statistically different with *p* < 0.05. *D*, heat maps showing temporal (*left*) and dose response (*right*) changes to glucose in INS-1 metabolite levels. Levels expressed as fold change *versus* time 0. For temporal response, cells were incubated in KRHB with 0.5 mm glucose for 30 min then stimulated to 10 mm glucose and sampled over 45 min. For dose response (*right*) INS-1 cells were incubated in KRHB + 0.2% BSA and treated with a step change from 0.5 to the new glucose concentration for 30 min. For both heat maps, *asterisk* indicates significant difference in peak area *versus* time 0 with *p* < 0.05.

The EC_50_ value for insulin secretion from INS-1 832/13 cells in response to glucose was 6.2 mm with near maximal insulin secretion observed at ∼10 mm glucose (Fig. 1*A*), similar to previous reports (8). Insulin secretion rate following a step change from 0.5 to 10 mm glucose showed a relatively sharp peak at ∼4 min (28 ng/mg protein/min) and a smaller broad peak with maxima at ∼25 min corresponding to 1st phase and 2nd phase of insulin secretion (Fig. 1*B*, see also supplemental Fig. S2 for calculation of secretion rate), consistent with previous reports of GSIS in islets (14) and INS-1 832/13 cells (15).

Using LC-TOF-MS, a total of 1030 mass spectral features were detected in INS-1 832/13 cells at both 0.5 and 10 mm glucose. Following a step change from 0.5 to 10 mm glucose, 190 features showed statistically significant (*p* < 0.05) differences of at least 1.5-fold (Fig. 1*C*). The identity of 87 metabolites was determined by accurate mass search of the Human Metabolome Database, comparison of theoretical and observed isotopic distributions and, when available, coelution with authentic standards. A summary of the identified metabolites is given in supplemental Table S1.

These metabolites were quantified relative to baseline at multiple times following a step increase in glucose from 0.5 to 10 mm glucose (Fig. 1*D*). We also measured the effect of different glucose concentrations at a single time point (25 min). To determine if peak area changes were linearly related to concentration, we performed standard addition experiments for 26 metabolites (see “Experimental Procedures”). As shown in supplemental Fig. S1, responses were linear in the concentration range found suggesting that the peak area differences accurately reflect relative changes in concentration, thus matrix effects on ionization were low for these experiments. While we cannot rule out non-linear effects of matrix on some analytes, these results along with the observation that only a small fraction of the detected features actually changed with glucose, indicating a relative constant matrix, suggest that peak areas are a good measure of relative concentration change. This conclusion is in agreement with previous metabolomic study using LC-TOF-MS (16). To assess metabolite pool size, the absolute concentrations of 44 metabolites were measured by standard addition at 10 mm glucose for 30 min (supplemental Table S1).

##### Metabolites Associated with Modulation of K_ATP_ Channels

**FIGURE 2.**
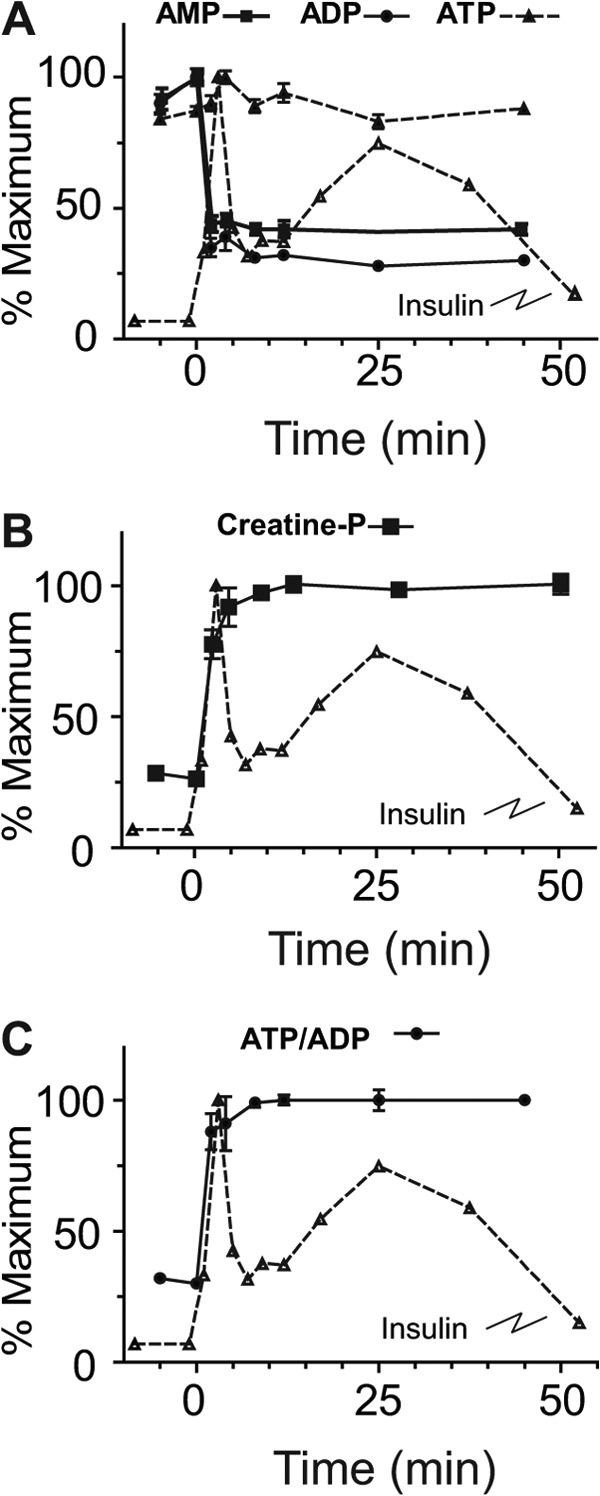
**Glucose-stimulated time course of adenine nucleotides.** INS-1 cells were treated with 10 mm glucose and sampled at the indicated time points from −5 to45 min. *A*, ATP, ADP, and AMP. *B*, phosphocreatine, *C*, ATP/ADP ratios. Insulin secretion rate is overlaid for comparison. Error bars represent 1 S.E., *n* = 3 for each time point.

K_ATP_ channel closure through rise in the ATP/ADP ratio is an established trigger for GSIS. In agreement with this concept, we detected a rapid increase in ATP/ADP ratio (Figs. 1*D* and 2*C*) that coincided with 1^st^ phase insulin release. This observation matches previous reports of a severalfold increase in ATP/ADP in mouse β-cells that reached a maximum 1 to 3 min following glucose stimulation (17) and a ∼2-fold increase in mouse islets within 5 min of glucose stimulation (18).

The absolute increases in ATP concentration were <15% while both ADP and AMP fell markedly (Figs. 1*D* and 2*A*) supporting the observations that K_ATP_ channel may be more influenced by reductions in ADP concentration (19) than increases in ATP. The ATP increase was limited by the availability of AMP and ADP which were only ∼10% of the ATP pool size at 0.5 mm glucose (supplemental Table S1). Hence, a maximal increase of ∼10% in ATP levels is possible from AMP and ADP substrate without *de novo* synthesis. These measurements do not take into account the apparent rapid ATP turnover indicated by rapid rise in the level of phosphocreatine (Figs. 1*D* and 2*B*). Phosphocreatine has been suggested to serve as a shuttle for energy rich phosphate from mitochondria to plasma membrane with metabolism by K_ATP_ channel-associated creatine kinase to phosphorylate ADP to ATP, increasing the local ATP concentration (20).

**FIGURE 3.**
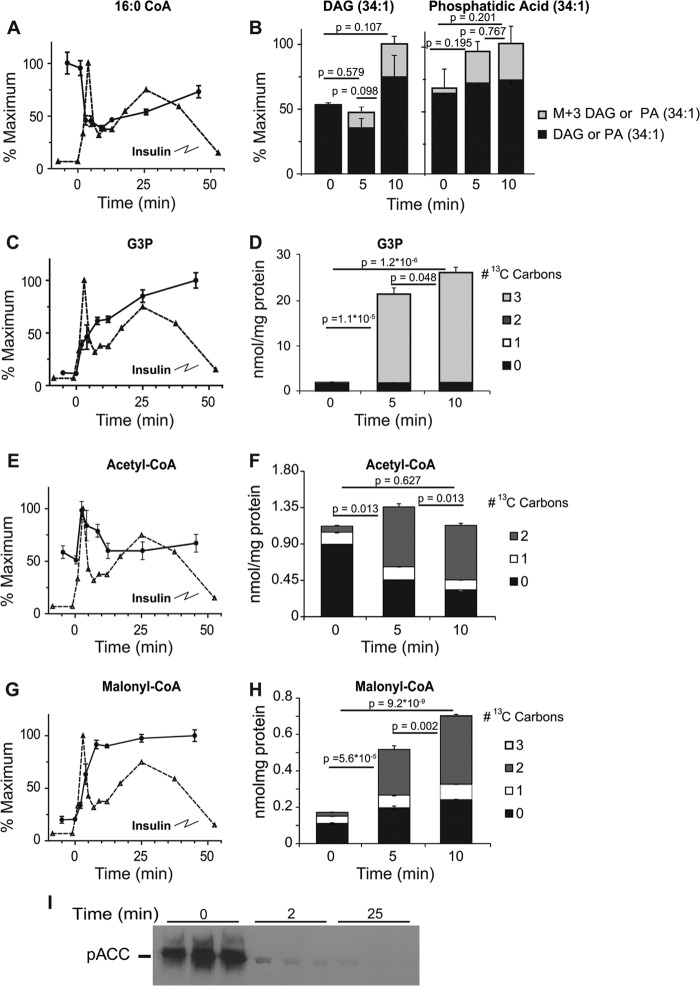
**Changes in metabolites associated with the malonyl-CoA mechanism of insulin secretion.** Time-dependent changes in the relative concentration of 16:0 acyl-CoA (*A*), glycerol-3-phosphate (*C*), acetyl-CoA (*E*), and malonyl-CoA (*G*) levels following stimulation with 10 mm glucose in INS-1 cells. Changes in total mass and ^13^C-labeled isotopologues of phosphatidic acid (*B*), glycerol-3-phosphate (*D*), acetyl-CoA (*F*), and malonyl-CoA (*H*) following stimulation with 10 mm U-[^13^C]glucose. Error bars represent 1 S.E., *n* = 3. *I*, levels of phosphorylated acetyl-CoA carboxylase (*pACC*) at baseline and following stimulation of INS1 cells with 10 mm glucose for 2 or 25 min. *p* values are given for total pool size on the graph. *p* values for isotopologue analysis are indicated in the text.

In addition to adenine nucleotides and phosphocreatine, long-chain acyl-CoAs have been proposed to modulate K_ATP_ activity such that decreases in concentration aid closure of K_ATP_ channels (21, 22). Previous studies have been discordant on the actual concentration changes of long-chain acyl-CoAs associated with glucose stimulation with both increases and decreases being reported (23, 24). Such differences may be due to cell treatment prior to glucose stimulation and time of analysis after glucose addition. We found an inverse dose-response relationship of long chain acyl-CoAs to glucose (Fig. 1*D*). The decrease in long-chain acyl-CoAs was rapid and concurrent with Phase 1 GSIS, *e.g.* 16:0-CoA decreased ∼50% within 2 min of glucose stimulation to ∼0.46 μmole/mg protein (Fig. 3*A*). Similar changes were observed in 14:0, 16:1, and 18:1 CoA upon glucose stimulation with time and in glucose dose-response profiles (Fig. 1*D*). To understand the fall in long chain-CoA levels, we measured diacylglycerol and phosphatidic acid. When INS-1 cells were stimulated with U-[^13^C]glucose, we found a non-significant trend for increases in phosphatidic acid and diacylglycerol species (Fig. 3*B*); however, we did observe a significant and rapid rise in a M+3 phosphatidic acid (34:1) 5 and 10 min after glucose addition (*p* = 9.4 × 10^−4^ and 2.3 × 10^−3^, respectively) and similar rises in diacylglycerol (34:1) M+3 isotopologues (*p* = 5.2 × 10^−4^ and 2.9 × 10^−3^, at 5 and 10 min, respectively) (Fig. 3*B*) suggesting addition of 3 carbons from [^13^C]glycerol-3-phosphate, which is rapidly generated by glycolysis (Fig. 3, *C* and *D*, see below). These results indicate that esterification, utilizing *de novo* generated glycerol-3-phosphate, facilitates removal of long chain acyl-CoAs from cytosol during the initial phase of glucose-stimulated insulin release. Such a decrease may be expected to aid closure of K_ATP_ channels, especially during first phase secretion (21, 22).

In addition to modulating K_ATP_ channel activity, long chain acyl-CoAs may also participate in additional downstream signaling events. One potential route is via the malonyl-CoA/long-chain acyl-CoA pathway in which glucose derived acetyl-CoA is carboxylated by acetyl-CoA-carboxylase (ACC) to form malonyl-CoA. Malonyl-CoA inhibits carnitine palmitoyl transferase 1 (CPT1) reducing fatty acid uptake and causing cytosolic accumulation of long-chain acyl-CoAs (or downstream metabolites). It has been proposed that the resulting long chain acyl-CoAs, or downstream metabolites, are important signaling molecules or coupling factors in secretory processes such as vesicular trafficking (25, 26).

The metabolomic data provide a means to assess the changes expected from this pathway. After stimulation with 10 mm glucose, acetyl-CoA doubles at 2 min followed by a gradual decrease to levels near baseline by 5 min (Figs. 1*D* and 3*E*). This decrease is likely due to rapid turnover of acetyl-CoA as evidenced by the decrease in ^12^C-labled and a significant increase (*p* = 3.4 × 10^−6^ and 3.5 × 10^−7^ at 5 and 10 min, respectively) in M+2 ^13^C-labeled species following stimulation with U-[^13^C]glucose (Fig. 3*F*). Similarly, there is an increase in total (Fig. 3*G*) and ^13^C-labeled malonyl-CoA (*p* = 2.3 × 10^−5^ and 5.5 × 10^−9^ at 5 and 10 min, respectively) (Fig. 3*H*) with only +2 *m*/*z* increasing, suggesting a rapid carboxylation of newly formed acetyl-CoA, likely derived via citrate and ATP-citrate lyase (see below) (27, 28). We also found near complete dephosphorylation/activation of ACC, after only 2 min of glucose exposure (Fig. 3*I*). The inhibition of CPT1 by malonyl-CoA may redirect acyl-CoAs to autonomous signaling events or condensation with *de novo* generated glycerol-3-phosphate, described above, to generate phosphatidic acid and diacylglycerols to participate in amplification of insulin secretion (29). This rapid increase in M+3 phosphatidic acid and diacylglycerol suggests that the generation of diacylglycerol for amplification not only comes from the activation of phospholipase (see Ref. 30 for discussion), but also from rapid generation via esterification of fatty acids. Thus, our results support a model in which glucose-derived glycerol-3-phosphate rapidly reacts with long-chain acyl-CoAs resulting in the removal of long chain acyl-CoAs, which, as pointed out above, could enhance closure of the K_ATP_ channel (21, 22) while simultaneously generate coupling factors for GSIS. Further, the malonyl CoA production supports the supply of long-chain CoA for this pathway, likely in addition to that derived from phospholipase activity of membrane phospholipids (31).

##### Glycolysis

Glycolysis, which comprises the first steps in glucose metabolism, supports the change in ATP/ADP ratio and closure of the K_ATP_ channel (32) and provides carbon for the pentose phosphate pathway (PPP) and TCA cycle. Hexose phosphates, comprised primarily of glucose-6-P and fructose-6-P (which are not resolved chromatographically) changed little over 45 min following stimulation with 10 mm glucose, increasing 1.2 fold over 8 min, then decreasing by 50% to ∼58 μmol/mg protein (Fig. 1*D* and supplemental Table S1). Dose-response studies show a 3-fold increase at 20 mm glucose (Fig. 1*D*). Rapid increase in concentrations of fructose bisphosphate, 2-phosphoglycerate with 3-phosphoglycerate and phosphoenolpyruvate (32-, 6.7-, and 5.5-fold, respectively) was observed following glucose stimulation (Fig. 1*D*), suggesting that phosphofructokinase is not rate-limiting to glycolysis in these cells.

Reoxidation of NADH to NAD^+^ is critical to maintaining glycolytic flux. The formation of glycerol-3-phosphate from dihydroxyacetone phosphate (DHAP) by cytosolic glycerol-3-phosphate dehydrogenase utilizes NADH to regenerate NAD^+^, allowing continuing flux through glycolysis. The proposed glycerol-3-phosphate shuttle posits a subsequent reoxidation of glycerol-3-phosphate to DHAP by mitochondrial glycerol-3-phosphate dehydrogenase, delivering NADH to the mitochondria (33). As indicated above, glycerol-3-phosphate increased 3.4-fold within 2 min of glucose stimulation (Fig. 3*C*). Stimulating cells with 10 mm U-[^13^C]glucose showed that the increase in glycerol-3-phosphate is due exclusively to *de novo* synthesis over the first 10 min of stimulation, demonstrated by the significant rise in an M+3 glycerol isotopologue (*p* = 1.1 × 10^−5^ and 2.2 × 10^−6^ at 5 and 10 min, respectively) (Fig. 3*D*). Our finding that there is a rapid esterification of fatty acids with *de novo* generated glycerol-3-phosphate (Fig. 3*B*), suggests that in addition to generating signaling intermediates, the esterification may also play a role in regeneration of cytosolic NAD^+^, enhancing glycolytic flux, thus maintaining a high ATP/ADP ratio.

##### TCA Cycle and Anaplerotic Shuttles

**FIGURE 4.**
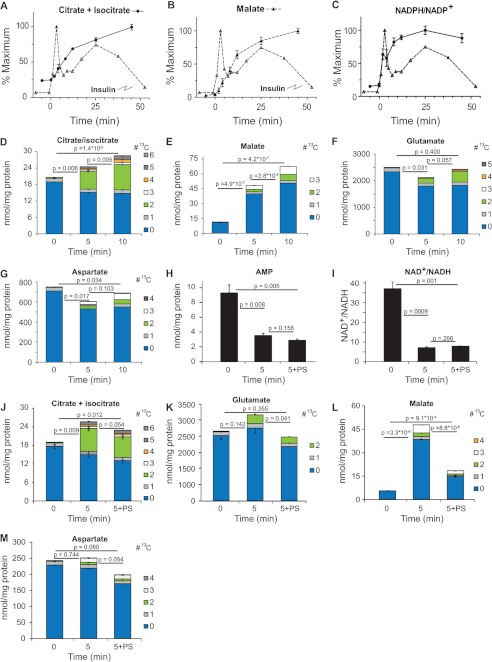
**Temporal changes of TCA cycle and related metabolites following addition of 10 mm glucose to INS-1 cells.** *A–D*, changes in the relative concentration of citrate + isocitrate (*A*), malate (*B*), and NADPH/NADP^+^ ratio (*C*). *D* and *E*, changes in total mass and ^13^C-labeled isotopologues of citrate + isocitrate (*D*), malate (*E*), glutamate (*F*), and aspartate (*G*) following stimulation with 10 mm U-[^13^C]glucose. *H–N*, effect of phenylsuccinate on metabolite levels. Cells were preincubated without or with 10 mm phenylsuccinate for10 min prior to addition of 10 mm glucose. Total metabolite concentration of AMP (*H*) and NAD/NADH ratio (*I*) and changes in relative concentration of ^13^C-labeled isotopologues of citrate + isocitrate (*D*), malate (*K*), glutamate (*L*), and aspartate (*M*). Error bars represent 1 S.D., *n* = 3. *Note 3*, *4*, and *5*, ^13^C-containing isotopologues of glutamate were obscured by presumptive breakdown products of phenylsuccinate. *p* values for isotopologue analysis are indicated in the text.

Metabolites in the TCA cycle participate in pathways that generate metabolites and cofactors which play a role in augmenting GSIS (3–5). The β-cell can increase TCA intermediates from pyruvate through pyruvate dehydrogenase and pyruvate carboxylase (34) and through additional anaplerotic reactions (35). As shown above, acetyl-CoA increased 1.5-fold within 2 min of stimulation and returned to prestimulation concentration within 12 min of glucose addition. Span 1 intermediates citrate + isocitrate (Figs. 1*D* and 4*A*) and α-ketoglutarate (Fig. 1*D*) increased 3–4-fold while, succinyl-CoA, and succinate showed much smaller absolute changes (Fig. 1*D*). The Span 2 metabolite malate, increased ∼30-fold after glucose stimulation (Figs. 1*D* and 4*B*).

To assess the mechanisms of change in TCA cycle metabolites, we measured their isotopic enrichment over the initial 10 min of exposure to 10 mm U-[^13^C]glucose. Acetyl-CoA is generated primarily from glucose as the M+2 isotopologue contributes to the majority of the *de novo* accumulated acetyl-CoA (*p* = 3.4 × 10^−6^ and 3.5 × 10^−6^, at 5 and 10 min, respectively) (Fig. 3, *E* and *F*) and is accompanied by a ∼40% rise in citrate + isocitrate, from 20 nmol/mg protein to 28 nmol/mg protein at 5 and 10 min (Fig. 4*D*). Approximately 90% of the increase in citrate mass was contributed by the M+2 isotopologue of citrate + isocitrate (*p* = 3.0 × 10^−6^) (Fig. 4*D*). The labeled pool continued to increase and after 10 min, the majority of the label as M+2 and less than 3% as M+3 or M+4 citrate + isocitrate. Thus, while total citrate + isocitrate levels rise slowly during the initial phases of insulin secretion, there is rapid turnover, with new citrate + isocitrate derived largely from pyruvate dehydrogenase (PDH) generated M+2 [^13^C]acetyl-CoA. The minimal increase in M+3 and M+4 isotopologues of citrate + isocitrate suggest that the majority of citrate + isocitrate exits the TCA cycle with minimal turns, at least in the early stages of GSIS.

Only a small percentage of glutamate is labeled with ^13^C in the first 10 min following glucose exposure (Fig. 4*F*). Importantly, there is a significant (*p* < 0.04) decrease in ^12^C-glutamate, about ∼20% of the pool, suggesting minimal anaplerotic flux into glutamate. Mitochondrial-derived glutamate has been proposed to participate in GSIS, in part through rapid accumulation of glutamate within the insulin secretory granules (36). While our data agree with previous studies (37), they do not support the hypothesis of rapid increases in intracellular glutamate during GSIS (38). We cannot rule out that the reduction in glutamate is due to uptake and subsequent release to the medium via secretory granules.

Although oxaloacetate could not be accurately measured because of its instability, we found a ∼3-fold increase in malate after 5 min and 4.8-fold after 10 min (Figs. 1*D* and 4*B*). A novel finding is that in cells exposed to 10 mm U-[^13^C]glucose, the observed increase in malate was largely accounted for by accumulation of [^12^C]malate, increasing the estimated concentration from 12 nmol/mg protein to 70 nmol/mg at 10 min, with only about 15% of the increase in mass due to *de novo* entry of ^13^C-label into malate (Fig. 4*E*). About 60% of this *de novo* derived [^13^C]malate is the M+3 isotopologue and ∼40% M+2 (Fig. 4*E*), suggesting near-equal generation from pyruvate carboxylase (M+3) and TCA cycle (M+2). The source of the unlabeled malate is likely the relatively large pool of aspartate (via oxaloacetate). The decrease in unlabeled aspartate is sufficient to account for the increase in unlabeled malate at 5 min (see Fig. 4, *E* and *G*). At 10 min, malate levels continue to rise and aspartate levels are restored to near baseline levels, with the increase due to accumulation of M+2 and M+3 isotopologues of aspartate in a similar proportion to that found in malate (Fig. 4*G*). This finding is consistent with a partial operation of the malate-aspartate shuttle, though we propose that the influx of aspartate is operating to provide anaplerotic oxaloacetate/malate to the TCA cycle and the transfer of NADH to the interior of the mitochondria. These results are consistent with the initial proposal by Simpson *et al.* (39) that aspartate is consumed during GSIS and forms the primary non-pyruvate carboxylase-derived anaplerotic substrate for the TCA cycle during GSIS. However, based on the isotopic labeling observed in the first 5–10 min of glucose treatment, our findings do not support the contention that the aspartate is derived from glucose (39) as there is minimal labeling of aspartate in the earliest time points following glucose addition.

The observed transient decrease in α-ketoglutarate concentrations in early time points following the addition of glucose (Fig. 1*D*) may be due to the transamination of α-ketoglutarate from aspartate to generate glutamate and oxaloacetate, with the latter subsequently reduced to malate, allowing the transfer of the glycolytically derived NADH to the mitochondria (see Fig. 7 for scheme). The glutamate can be converted back to α-ketoglutarate through glutamate dehydrogenase, with the generation of NAD(P)H and NH_4_^+^ as described previously (39).

To further support the role of the malate-aspartate shuttle in the rise in malate we pretreated INS-1 cells with phenylsuccinate, which inhibits the oxoglutarate carrier, to reduce efflux of α-ketoglutarate and entry of malate into the mitochondria (9). Pretreatment with phenylsuccinate did not impair reduction in AMP levels (Fig. 4*H*) or reduction in NAD^+^/NADH ratio (Fig. 4, *H* and *I*) following glucose stimulation. The rise in citrate (Fig. 4*J*) was blunted and glutamate levels fell (Fig. 4*K*) following phenylsuccinate treatment and this was largely due to changes in the levels of ^12^C-labeled isotopologues. There was a marked and significant reduction in the rise in malate following phenylsuccinate treatment (Fig. 4*L*), primarily in the ^12^C-label isotopologue. We suggest that the fall in malate is due to consumption of mitochondrial malate without replenishment by cytosolic malate. Aspartate levels are likewise reduced by phenylsuccinate treatment and, as with malate, the ^13^C-isotopologue distribution is not changed (Fig. 4*M*).

As inhibition of the oxoglutarate carrier inhibits glucose-induced insulin release (42), these data further confirm that malate-supported anaplerosis and/or shuttling of reducing equivalents by malate into the mitochondria are critical to sustaining insulin secretion. The observed rapid increase in malate can also clarify data by Lu *et al.* (37) who, through modeling of NMR-derived spectra of INS-1 cells extracts, proposed the presence of two distinct pools of pyruvate that enter into the Krebs cycle following glucose stimulation of β-cells, one derived from glycolysis and the other from a non-glycolytically derived pool. We believe our finding of a rapid rise in malate derived from a non-glucose source could, via cytosolic malic enzyme, generate pyruvate, giving rise to the second pool of pyruvate. Alternatively, the labeling pattern of highly glucose-responsive INS-1 cells lines presented by Lu *et al.*, may be explained by rapid increase in mitochondrial malate, via import into the mitochondria which would also result in a relative dilution of the glutamate pool. We favor the latter explanation as phenylsuccinate treatment results in a significant reduction in the ^12^C-isotopologue concentration of glutamate with minimal change in the M+2 ^13^C-isotopologue levels, presumably derived from PDH derived M+2 [^13^C]acetylCoA (Fig. 4*K*).

##### NADPH

NADPH has been cited as a critical metabolite for GSIS (1, 41). NADPH increased slightly (1.3-fold) but insignificantly over ∼12 min of glucose stimulation which is similar in magnitude to changes reported in islets (43) (Fig. 1*D*). The NADPH/NADP^+^ ratio increased abruptly after addition of glucose and increased 5.8-fold over 25 min, correlating well with 1st and 2nd phase insulin secretion (Figs. 1*D* and 4*C*). It has been proposed that cycling of metabolites from mitochondria to the cytosol generates malate, which can undergo oxidative decarboxylation to pyruvate and generation of NADPH, augmenting GSIS (1, 4, 40). This can occur directly from malate exported to the cytosol or from citrate-from ATP-citrate lyase generation of oxaloacetate which subsequently forms cytosolic malate or finally from isocitrate efflux through the citrate/isocitrate carrier where oxidized to ketoglutarate (α-KG) by NADP-dependent isocitrate dehydrogenase. The relatively rapid rise in M+2 malonyl-CoA isotopologue (Fig. 3*H*), suggests a significant flux of citrate from the mitochondria which generates M+2 acetyl-CoA (Fig. 3*F*) via ATP-citrate lyase (44) serving as a substrate for ACC to generate the malonyl-CoA. Unfortunately, interference by other metabolites makes it difficult to accurately quantify α-KG isotopomers higher than M+2 to estimate the isocitrate → α-KG contribution to generation of NADPH. Further studies will be needed to define which pathways contribute to the generation of NADPH.

##### Succinate and Short Chain Fatty Acids

**FIGURE 5.**
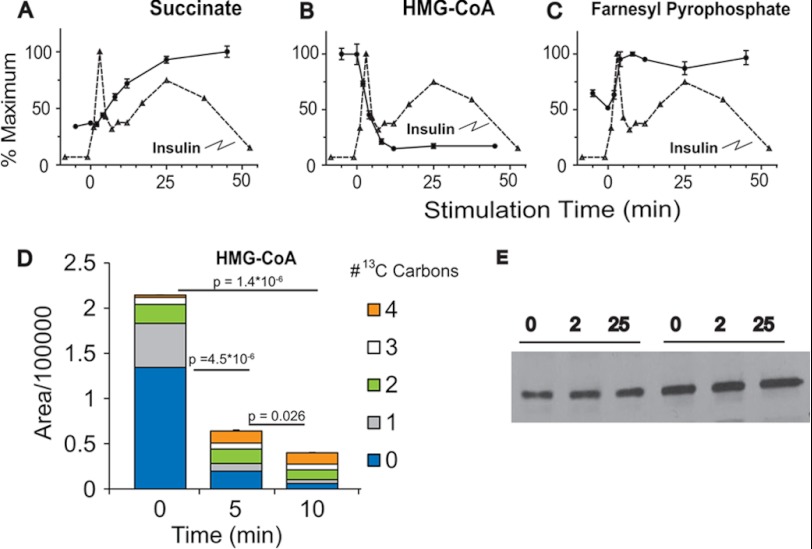
**Metabolites participating in the succinate mechanism of glucose-stimulated insulin secretion.** *A–C*, time-dependent changes in succinate (*A*), HMG-CoA (*B*), and farnesyl pyrophosphate (*C*). *D*, changes in total mass and ^13^C-labeled isotopologues of HMG-CoA following stimulation with 10 mm U-[^13^C]glucose. Error bars represent 1 S.E., *n* = 3. *E*, phosphorylated HMG-CoA reductase at baseline and following stimulation with glucose for 2 or 25 min. *p* values for isotopologue analysis are indicated in the text.

Succinate has been proposed as a key metabolite that participates in the generation of molecules that can act as second messengers for potentiation of insulin secretion (41) including short chain fatty acids, acetoacetate, malonyl-CoA, and 3-hydroxy-3-methyl-glutaryl-CoA (HMG-CoA) (41, 45). Following glucose exposure, we found a rapid increase in succinate levels (Figs. 1*D* and 5*A*) and malonyl-CoA (Figs. 1*D* and 3*H*) in the INS-1 832/13 cells. In contrast we found a significant reduction in HMG-CoA (Figs. 1*D* and 5*B*), similar to previous reports (46). The reduction in HMG-CoA levels appears to be due to rapid consumption as cells incubated for 5 or 10 min with U-[^13^C]glucose showed a rapid decrease in fully ^12^C-labeled HMG-CoA isotopologue and an increase in various ^13^C-labeled isotopologues suggesting *de novo* synthesis that cannot keep up with consumption (Fig. 5*D*). The reduction in HMG-CoA is apparently not due to changes in HMG-CoA reductase activity as we do not observe a change in the phosphorylation state of HMG-CoA reductase following glucose stimulation of INS-1 832/13 cells (Fig. 5*E*).

Farnesyl pyrophosphate, a downstream product of the HMG-CoA pathway involved in isoprenylation of proteins (47), was found to increase rapidly following glucose stimulation (Fig. 5*C*) in a time course that mirrors that of the fall in HMG-CoA and 1st phase insulin secretion. Recent studies have suggested that specific small G-proteins (Cdc42 and Rac1) play an important role in GSIS (48, 49). These signaling proteins undergo prenylation at their C-terminal cysteine residues which is essential for the transport and fusion of insulin-containing secretory granules with the plasma membrane and the exocytosis of insulin which has been implicated in GSIS (48). The rapid increase in substrate for isoprenylation, farnesyl pyrophosphate, suggests that such modifications can occur in a time frame to be relevant for 1st phase insulin secretion. Further, the dramatic reduction in HMG-CoA suggests that it may become limiting to flux through this pathway and therefore limiting GSIS.

##### PPP-derived Metabolites

Metabolites in the PPP are not often measured in investigations of GSIS but play a key part in cellular metabolism by supplying 5-carbon substrates for purine, pyrimidine, and histidine synthesis and generating NADPH for lipid biosynthetic pathways. However, the PPP is not highly active in β-cells and likely contributes little to the generation of NADPH (40). We observed that most PPP metabolites increased in parallel to increasing glucose concentration (Fig. 1, *A* and *B*). Rapid and substantial relative increases in the pentose phosphate pathway metabolites pentose phosphates, 6-phosphogluconate, sedoheptulose phosphates, and phosphoribosyl pyrophosphate (1.8-, 3.2-, 2.4-, and 7.4- fold increases, respectively) were observed. Significant labeling of 6-phosphogluconate following stimulation of cells with U-[^13^C]glucose was observed (not shown), indicating direct flux into the PPP. Although large relative increases in PPP metabolite levels with glucose stimulation are detected, the pool size of these metabolites is substantially smaller than for those of the TCA cycle (supplemental Table S1) supporting previous findings that the bulk of glucose carbon enters the TCA cycle and does not enter the PPP (50) and a relative unimportance for this pathway in the generation of NADPH in supporting GSIS.

**FIGURE 6.**
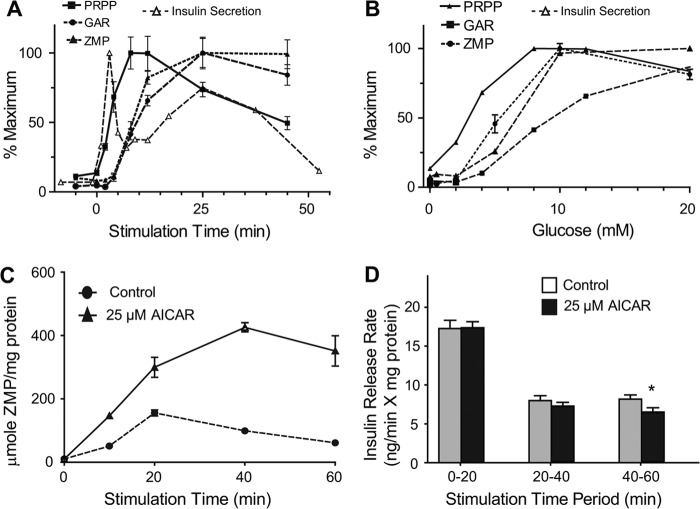
**Formation and effect of ZMP in INS-1 cells.** *A* and *B*, time (*A*) and concentration-dependent (*B*) changes in purine metabolic pathway in INS-1 cells stimulated with glucose. *PRPP*, phosphoribosylpyrophosphate, *GAR*, glycinamide ribotide, *ZMP*, 5-amino-4-imidazolecarboxamide ribotide. *C*, time-dependent formation of ZMP in INS-1 cells stimulated with 10 mm glucose with or without 25 μm AICAR. Error bars represent 1 S.E., *n* = 3. *D*, insulin secretion rate measured by change in incubation buffer insulin concentration over indicated time period. Error bars represent 1 S.E., *n* = 8. *Asterisk* indicates significant difference in insulin release rate with *p* < 0.05.

ZMP is an endogenous metabolite in the purine synthesis pathway and a precursor to IMP. Although measurements of endogenous ZMP levels have not been reported in β-cells, we detected a 9-fold increase to ∼4.0 μmol/mg protein that reached a maximum ∼25 min after glucose stimulation (Figs. 1*D* and 6*A*). Supporting an increase by *de novo* biogenesis as opposed to stabilization of ZMP, we found that both phosphoribosyl pyrophosphate which links the PPP to the nucleotide synthesis pathway and glycinamide ribotide, a ZMP precursor, were also detected and increased both temporally and in a dose-response manner after addition of glucose (Figs. 1*D* and 6, *A* and *B*).

ZMP can substitute for AMP in enhancing phosphorylation and activation of AMP-activated protein kinase (AMPK), an important regulator of cellular energy balance (51). Indeed, AICA riboside (AICAR), which is phosphorylated in cells to generate ZMP, is widely used to activate AMPK. Acute exposure to AICAR has been found to both decrease (52, 53) and potentiate insulin release (54). Based on the timing of the increase in endogenous ZMP found in our studies, we hypothesized that it may serve as a negative regulator of GSIS during the second phase of insulin release. To test this idea, we treated INS-1 832/13 cells with 10 mm glucose and 25 μm AICAR and achieved ∼4× higher intracellular ZMP levels at 40 min relative to control cells stimulated with 10 mm glucose only (Fig. 6*C*). This increase in ZMP was accompanied by 20% reduction in rate of insulin release 40 to 60 min post glucose stimulation (Fig. 6*D*). Although ZMP increases, there was no evidence of activation of AMPK as no change was seen in the phosphorylation of HMG-CoA reductase and a decrease in ACC-1 phosphorylation was observed following glucose addition (Fig. 3*I*). Therefore, while ZMP may restrain GSIS, at endogenous levels this effect does not seem to be through AMPK; perhaps through an alternate route such as altering lipid metabolism independent of AMPK activation (55).

##### Sugar Nucleotide Donors

We detected 8 common sugar nucleotide donors with GDP-mannose changing the most substantially (Fig. 1*D*). GDP-mannose forms from conversion of glycolytic intermediate fructose-6-phosphate to mannose-6-phosphate and mannose-1-phosphate before condensing with GTP (56). This metabolite has not previously been quantified in β-cells; but, we observe a rapid increase that peaked at 14-fold over basal (∼4.2 μmol/mg protein) within 8 min of glucose followed by a gradual decrease. GDP-fucose, a product of GDP-mannose metabolism (57), was unchanged. While determination of these metabolites in β-cells have not been previously explored, we have previously described that activation of the insulin-like growth factor II (IGF-II)/mannose-6-phosphate (M-6-P) receptor by IGF-II results in augmentation of insulin secretion, even at low concentrations of glucose (58). The binding of mannose-6-phosphate-tagged proteins may play a regulatory role in insulin secretion by interaction with insulin secretory vesicles.

##### Additional Metabolites

In these studies, we identified additional metabolites that showed dynamic changes following glucose exposure. In addition to adenosine, other mono- and diphosphonucleotides decreased by 1.7–4.3-fold within 2 min of glucose stimulation. GTP increased only slightly (< 5%) whereas UTP and CTP increased 40 and 80%, respectively with reciprocal falls in their mono and dinucleotides (Fig. 1*D*). Recent studies have provided evidence for a significant role for mitochondrial GTP in GSIS (59). While GTP levels do not change significantly, the pool of mitochondrial GTP is small compared with the cytosolic pool, thus changes in the mitochondrial pool may not be detectable. The role of UTP and CTP in GSIS remains to be determined.

We also observed decreases in pantothenic acid with glucose. As this metabolite is important for CoA synthesis, its decrease may reflect consumption in *de novo* production of CoAs. Besides long-chain acyl-CoAs and farnesyl pyrophosphate discussed above, we also found changes in other compounds involved in lipid metabolism including decreases in free fatty acids and citicolline, an intermediate in production of phosphatidylcholine. The decrease in intracellular free fatty acid content may be due to secretion or to consumption for production of other lipid signaling molecules. While investigation of all these pathways is beyond the scope of this report, these results indicate that this method may be used for studying a wide range of metabolites and pathways connected to insulin secretion.

## CONCLUSIONS

**FIGURE 7.**
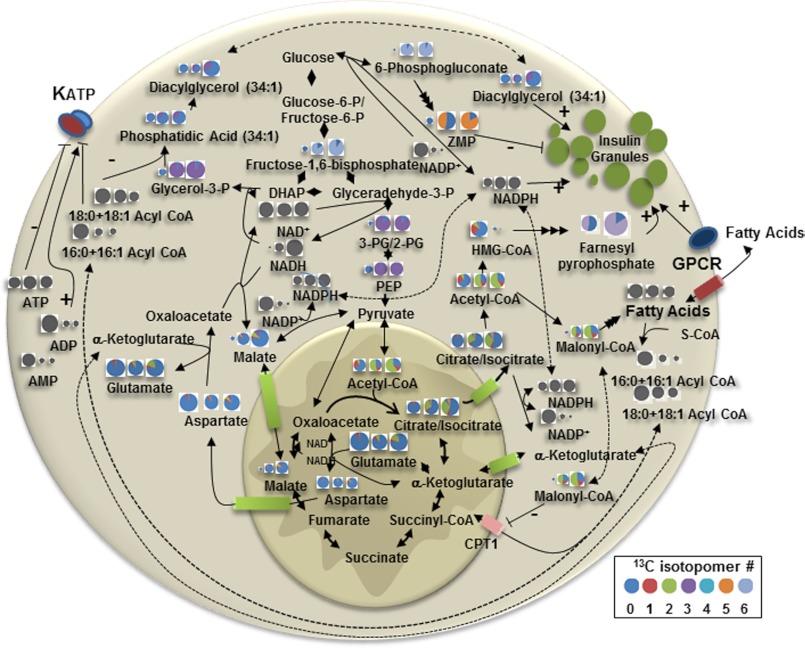
**Schematic of metabolite levels and ^13^C enrichment of indicated metabolites at baseline and after 5 and 10 min exposure to 10 mm glucose.** The size of each circle denotes the relative concentration of that metabolite, but does not reflect the relative concentration among the metabolites. ^13^C isotopologue percentage is indicated by the gradation of the pie and includes the baseline natural enrichment of ^13^C of ∼1%. *Gray circles* indicate that the isotopic enrichment was not determined for those metabolites.

The use of LC-TOF-MS has allowed us to measure the temporal and dose response to increasing glucose concentration for a wide range of metabolites in INS-1 832/13 cells. By combining static (Fig. 1) measurements and flux analysis (Figs. 3–5) we have confirmed, extended or proposed modifications to several prevailing hypotheses regarding the metabolic pathways associated with GSIS (Fig. 7). The simultaneous measurement of glycolytic and TCA cycle intermediates, nucleotides, long- and short-chain acyl-CoAs and other intermediates show a novel interaction of metabolites. Novel findings include: 1) rapid esterification of long chain acyl-CoA with *de novo* synthesized glycerol-3-phosphate which explains the rapid fall in acyl-CoAs, allowing enhanced closure of the K_ATP_ channel and generation of phosphatidic acid and diacylglycerols to act as possible second messengers. 2) Rapid turnover of Span 1 TCA metabolites and rapid increases in the Span 2 metabolite, malate, which increases primarily by influx of malate into the mitochondria. 3) Rapid generation of malonyl-CoA from citrate coincident with a glucose-induced dephosphorylation of ACC1. 4) Significant flux of glucose-derived carbon into HMG-CoA and a rapid depletion of this metabolite with a parallel, rapid rise in farnesyl pyrophosphate, providing substrate for isoprenylation of proteins. 5) *De novo* synthesis of ZMP from glucose entering the pentose phosphate pathway which may modulate insulin release. 6) Increases in sugar nucleotides, including GDP-mannose, which may provide substrate for modification of proteins important for secretion.

These studies are limited to the glucose-responsive INS1 832/13 cells but many findings in these cells have been shown to occur in islets derived from rodents. The studies presented here provide several hypotheses for metabolic pathways that may play varying roles in the augmentation of GSIS which can be tested in additional experiments in both INS-1 cells and in isolated isles. Assessing the alterations of specific metabolites in response to high glucose or fatty acid exposure may also provide information as to how chronic insulin resistance associated with obesity may result in altered β-cell metabolism which may lead to altered insulin secretory dynamics and diabetes mellitus.

## Supplementary Material

Supplemental Data

## Footnotes

3 The abbreviations used are:

GSIS: glucose-stimulated insulin secretion
ZMP: 5-aminoimidazole-4-carboxamide ribotide
TCA: tricarboxylic acid
AICAR: 5-amino-1-β-d-ribofuranosyl-imidazole-4-carboxamide riboside
TOF-MS: time-of-flight mass spectrometry
ESI: electrospray ionization
ACC: acetyl-CoA carboxylase
PPP: pentose phosphate pathway
AMPK: AMP-activated protein kinase.
