# Supplementary material for: Metabolome Response to Glucose in the β-Cell Line INS-1 832/13
Source: J Biol Chem. 2013 Feb 20;288(15):10923–35. doi: 10.1074/jbc.M112.414961 (PMC3624472; doi:10.1074/jbc.M112.414961)
Supplement: Supplemental Data [file supp_288_15_10923__index.html]

Metabolome Response to Glucose in the β-Cell Line INS-1 832/13 — β-Cell Metabolomics — Supplemental Data 

# Metabolome Response to Glucose in the β-Cell Line INS-1 832/13

## Supplemental Data

**Files in this Data Supplement:**

- Supplemental figures 1 and 2 and supplemental table 1 (.pdf, 198 KB) - Supplemental figures and table
