# Supplementary material for: Metabolome Response to Glucose in the β-Cell Line INS-1 832/13
Source: J Biol Chem. 2013 Feb 20;288(15):10923–35. doi: 10.1074/jbc.M112.414961 (PMC3624472; doi:10.1074/jbc.M112.414961)
Supplement: Supplemental Data [file supp_M112.414961_jbc.M112.414961-1.pdf]

Supplemental Information for

Metabolome Response to Glucose in the β-cell line INS-1 832/13

Matthew A. Lorenz[1,4], Mahmoud A. El Azzouny[1,4], Robert T. Kennedy[1,2] Charles F. Burant[3]

Figure and Table Captions for Supporting Information:

Supplemental Figure 1. Results from standard addition experiment to determine linearity of response for 26 metabolites in cell extract matrix. An extract of INS-1 cells that had been incubated for 2 h in 0.5 mM glucose in KRB was divided into aliquots. The aliquots were spiked to create different concentrations of 24 metabolites and a constant final volume of extract. Each sample was analyzed by LC-TOF-MS and peak areas plotted against spiked concentration (i.e., does not incorporate endogenous concentration). Analytes that have a smaller number of points in the lines have high concentrations so low concentration spikes were not used because they had small effects on peak area. Each plot indicates the R^2 value from linear regression analysis.

Supplemental Figure 1.

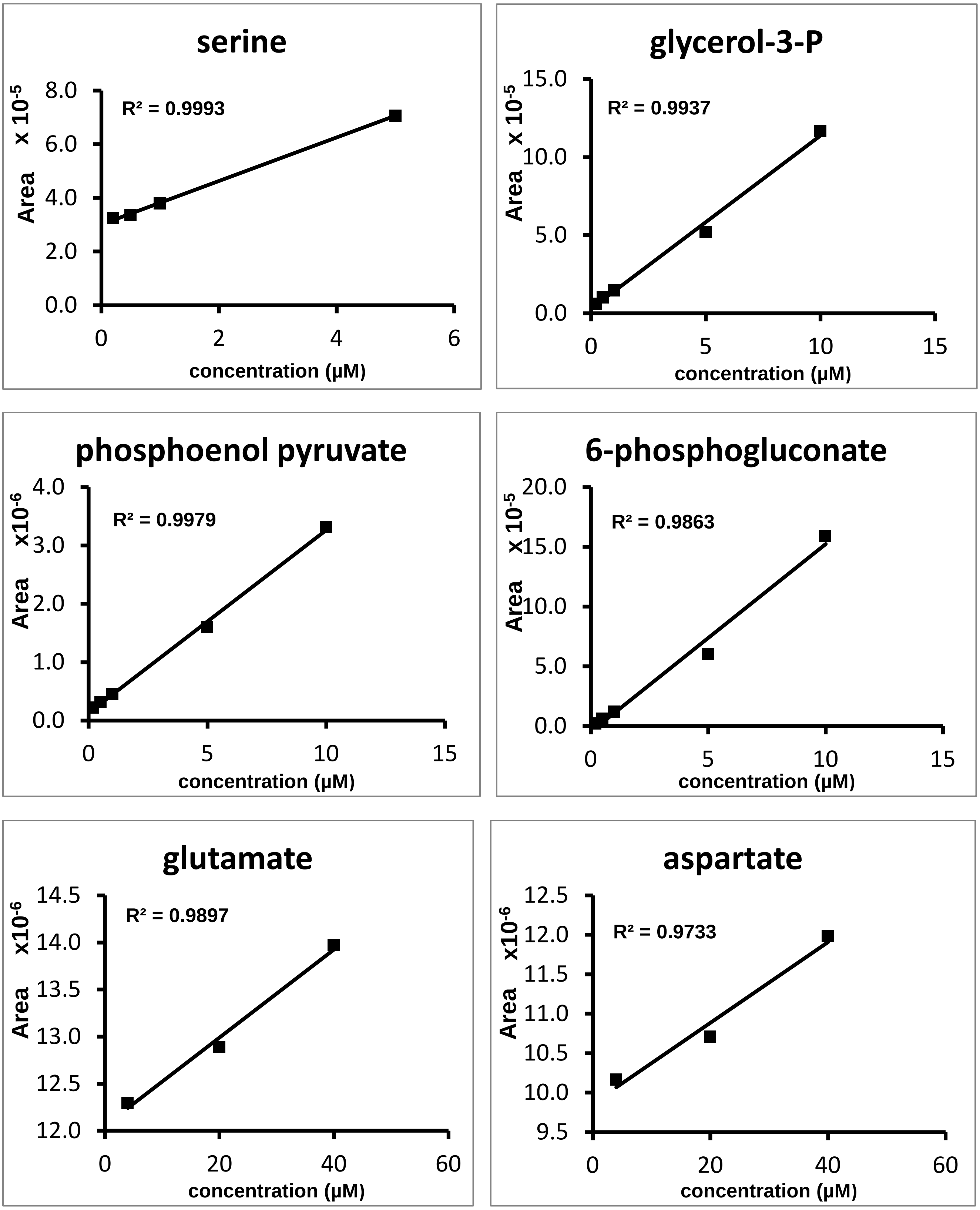

Supplemental Figure 1 (continued).

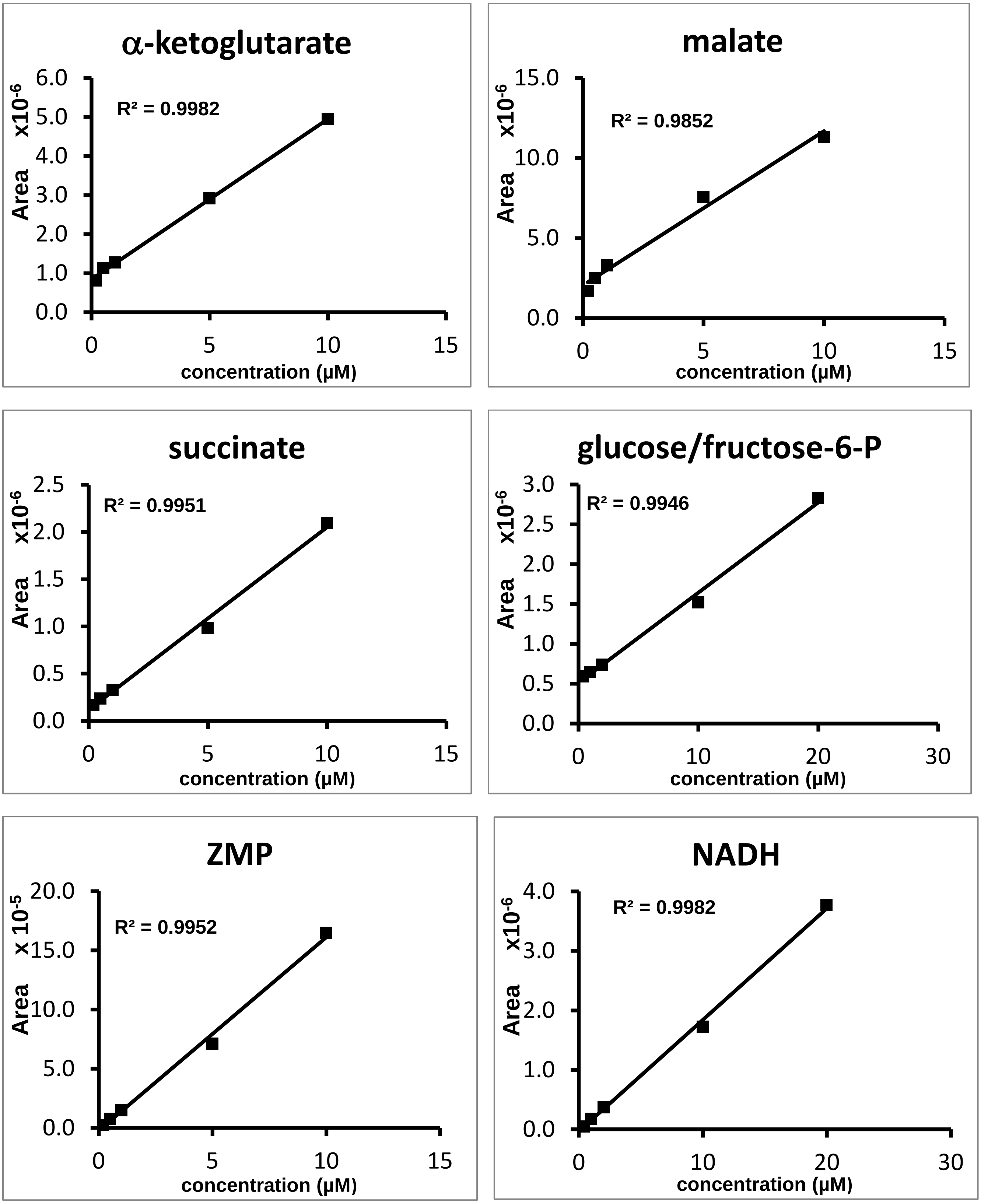

Supplemental Figure 1 (continued).

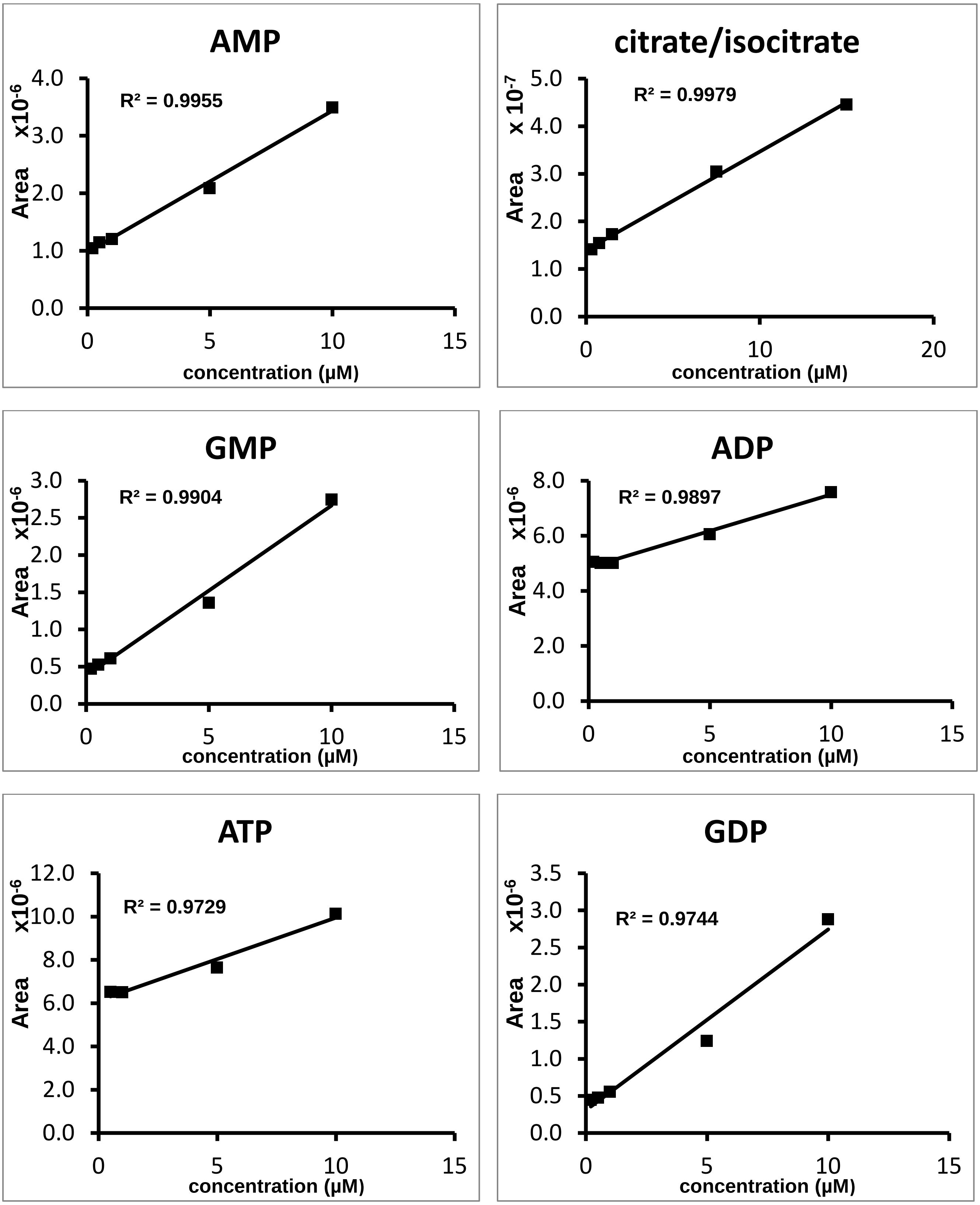

Supplemental Figure 1 (continued).

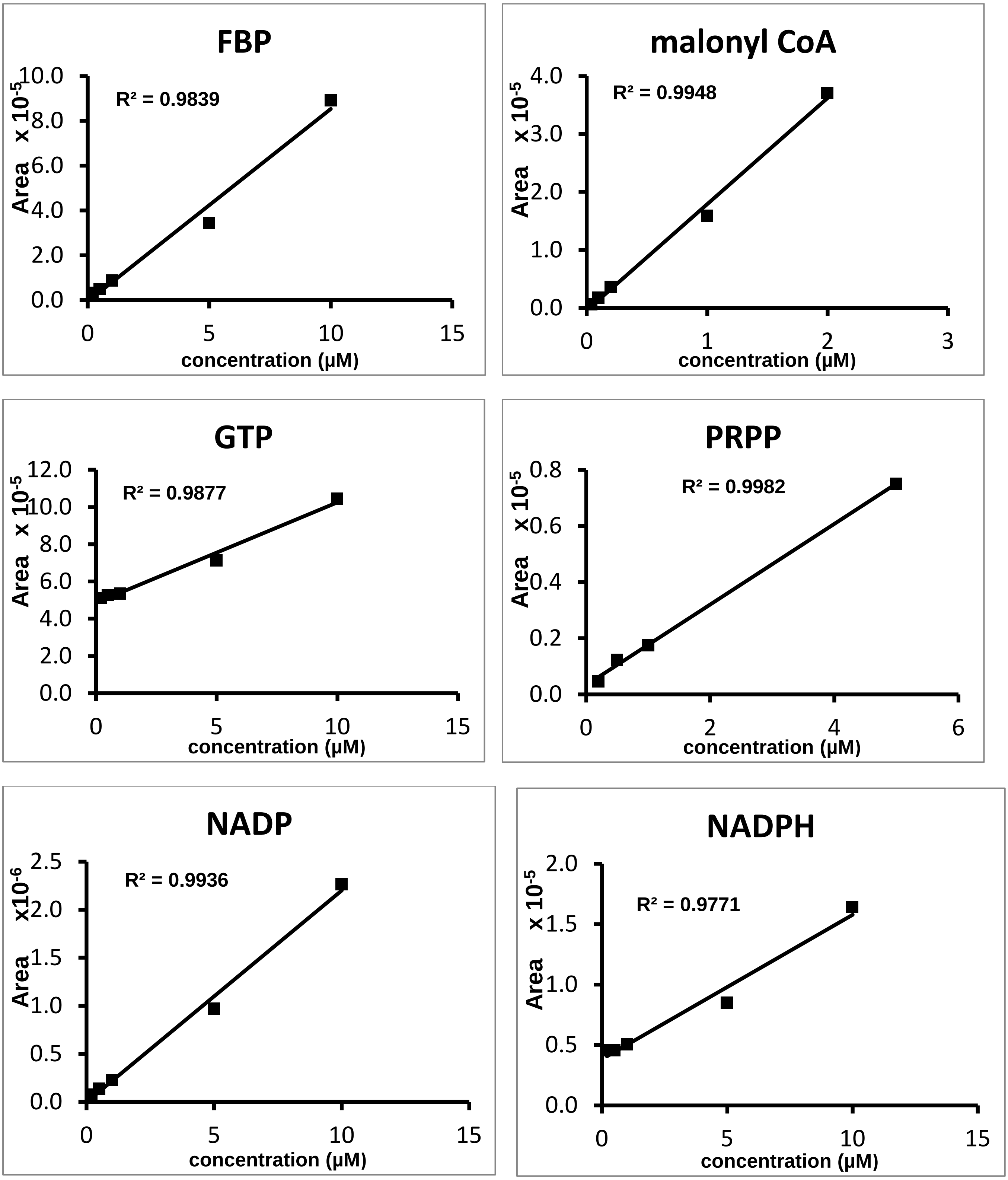

Supplemental Figure 1 (continued).

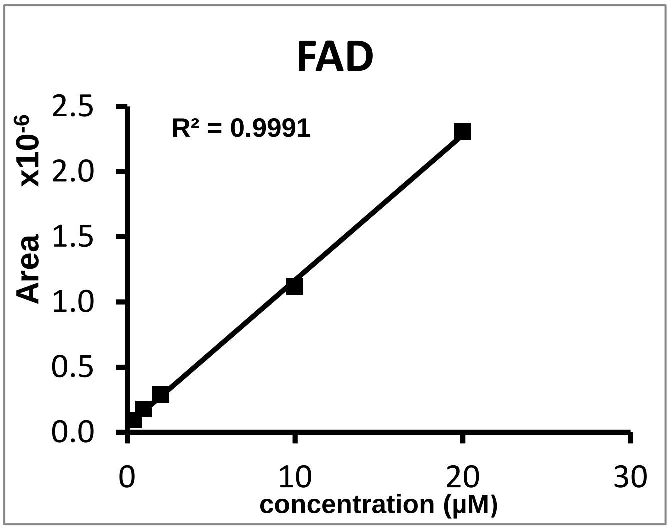

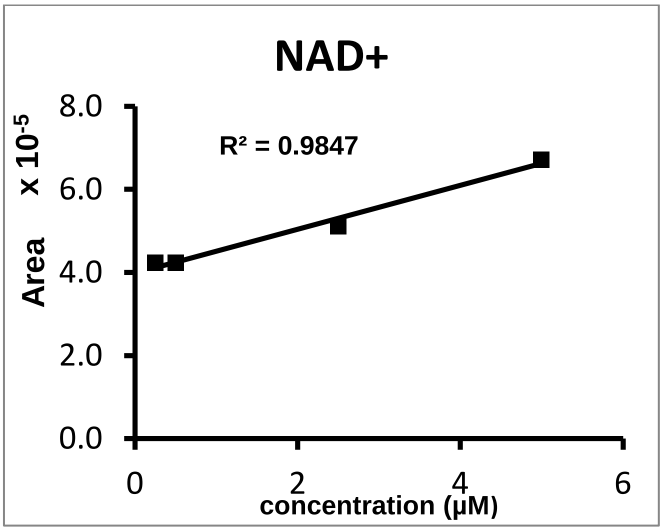

Supplemental Figure 2. Insulin secretion rate data for INS-1 cells. (a) Total insulin secreted versus stimulation time (0.5 to 10 mM glucose at time 0) for 3 plates of INS-1 cells (a, b and c). Error bars represent SEM of analytical replicates, n = 2. (b) Insulin secretion rate versus stimulation time. Average secretion rate was calculated as the difference in insulin released between each sampling interval and plotted (blue dash). The average rate during the sampling interval is plotted in red.

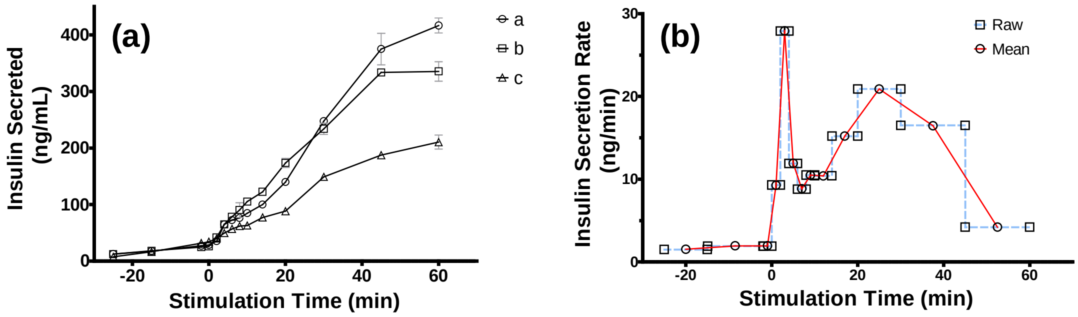

Supplemental Table 1. Summary of metabolites identified and quantified by LC-TOF-MS in INS-1 cells. Metabolites were identified by matching the detected mass to the Human Metabolome Database, comparing isotope ratios expected to observed, and by matching retention time with authentic standards where standards were available. Selected metabolites were quantified by the standard addition method to INS-1 extracts to determine their pool size. Authentic standards were added to sample at 3 different concentrations for the standard addition work.

Table 1.

Metabolite	Abbreviation	Identification (ID) method[1]	Molecular Formula	^{13}C - Internal Standard Used?	Theoretical Mass (amu)	Measured Mass (amu)	Difference between theoretical and measured mass (ppm)	Concentration[2] (nmole/mg protein)
**Glycolysis**								
hexose-phosphate	HP	S	$C_6H_{13}O_9P$	Yes	260.0297	260.0289	-3.0	58
fructose 1,6-bisphosphate	FBP	S	$C_6H_{14}O_{12}P_2$	Yes	339.9960	339.9956	-1.1	13
2-phosphoglycerate + 3-phosphoglycerate	2PG+3PG	S	$C_3H_7O_7P$	No	185.9929	185.9924	-2.6	11
phosphoenolpyruvate	PEP	S	$C_3H_5O_6P$	Yes	167.9824	167.9812	-7.0	2.3
acetyl- Coenzyme A	aCoA	S	$C_{23}H_{38}N_7O_{17}P_3S$	No	809.1258	809.1242	-1.9	1.6
glycerol-3-phosphate	G3P	S	$C_3H_9O_6P$	No	172.0137	172.0135	-1.0	15
lactate	LAC	S	$C_3H_6O_3$	No	90.0317	90.0315	-2.0	41
**Tricarboxylic Acid Cycle**								
citrate+isocitrate	CIT+ICIT	S	$C_6H_8O_7$	Yes	192.0270	192.0262	-4.1	110
alpha-ketogluterate	AKG	S	$C_5H_6O_5$	Yes	146.0215	146.0207	-5.3	22
succinyl-coa	sCoA	S	$C_{25}H_{40}N_7O_{19}P_3S$	No	867.1312	867.1294	-2.0	0.85
succinate	SUC	S	$C_4H_6O_4$	Yes	118.0266	118.0275	7.8	22
malate	MAL	S	$C_4H_6O_5$	No	134.0215	134.0211	-2.8	67
malonyl- Coenzyme A	mCoA	S	$C_{24}H_{38}N_7O_{19}P_3S$	No	853.1156	809.1208	-6.1	0.86
aspartic acid	Asp	S	$C_4H_7NO_4$	No	133.0375	133.0372	-2.1	750
glutamic acid	Glu	S	$C_5H_9NO_4$	No	147.0532	147.0528	-2.6	2500
**Pentose phosphate pathway**								
ribose phosphate	R5P	S	$C_5H_{11}O_8P$	No	230.0191	230.0183	-3.4	3.4
6-phosphogluconic acid	6PG	S	$C_6H_{13}O_{10}P$	No	276.0246	276.0242	-1.4	2.7
sedoheptulose-7-phosphate	S7P	S	$C_7H_{15}O_{10}P$	No	290.0403	290.0405	0.8	12
phosphoribosyl pyrophosphate	PRPP	S	$C_5H_{13}O_{14}P_3$	No	389.9518	389.9505	-3.3	3.5
**Nucleotides**								
adenosine monophosphate	AMP	S	$C_{10}H_{14}N_5O_7P$	Yes	347.0631	347.0620	-3.1	11
adenosine diphosphate	ADP	S	$C_{10}H_{15}N_5O_{10}P_2$	No	427.0294	427.0282	-2.8	18
adenosine triphosphate	ATP	S	$C_{10}H_{16}N_5O_{13}P_3$	Yes	506.9957	506.9949	-1.5	850
guanosine monophosphate	GMP	S	$C_{10}H_{14}N_5O_8P$	No	363.0580	363.0577	-0.8	2.8
guanosine diphosphate	GDP	S	$C_{10}H_{15}N_5O_{11}P_2$	No	443.0243	443.0245	0.5	4.5
guanosine triphosphate	GTP	S	$C_{10}H_{16}N_5O_{14}P_3$	Yes	522.9907	522.9900	-1.3	3.9
uridine monophosphate	UMP	S	$C_9H_{13}N_2O_9P$	No	324.0359	324.0350	-2.7	na
uridine diphosphate	UDP	S	$C_9H_{14}N_2O_{12}P_2$	No	404.0022	404.0020	-0.4	na
uridine triphosphate	UTP	S	$C_9H_{15}N_2O_{15}P_3$	No	483.9685	483.9679	-1.2	na
cytidine monophosphate	CMP	S	$C_9H_{14}N_3O_8P$	No	323.0518	323.0502	-4.9	na
cytidine diphosphate	CDP	S	$C_9H_{15}N_3O_{11}P_2$	No	403.0182	403.0178	-0.9	na
cytidine triphosphate	CTP	S	$C_9H_{16}N_3O_{14}P_3$	No	482.9845	482.9833	-2.4	na
nicotinamide adenine dinucleotide	NAD	S	$C_{21}H_{27}N_7O_{14}P_2$	No	663.1091	663.1089	-0.3	37
nicotinamide adenine dinucleotide, reduced	NADH	S	$C_{21}H_{28}N_7O_{14}P_2$	No	665.1242	665.1240	-0.3	2.0
nicotinamide adenine dinucleotide phosphate	NADP	S	$C_{21}H_{28}N_7O_{17}P_3$	No	743.0755	743.0710	-6.0	0.92
nicotinamide adenine dinucleotide phosphate, reduced	NADPH	S	$C_{21}H_{29}N_7O_{17}P_3$	No	745.0911	745.0925	1.9	4.0
flavin adenine dinucleotide	FAD	S	$C_{27}H_{33}N_9O_{15}P_2$	No	785.1571	785.1565	-0.7	2.6
**Amino Acids**								
asparagine	Asn	S	$C_4H_8N_2O_3$	No	132.0535	132.0531	-2.9	39
glutamine	Gln	S	$C_5H_{10}N_2O_3$	No	146.0691	146.0687	-2.6	76
lysine	Lys	S	$C_6H_{14}N_2O_2$	No	146.1055	146.1050	-3.3	15
ornithine	Orn	S	$C_5H_{12}N_2O_2$	No	132.0899	132.0895	-2.9	22
serine	Ser	S	$C_3H_7NO_3$	No	105.0426	105.0421	-4.6	25
**Long Chain CoAs**								
14:0 Conenzyme A	14:0-CoA	D	$C_{35}H_{62}N_7O_{17}P_3S$	No	977.3135	977.3108	-2.7	na
16:0 Conenzyme A	16:0-CoA	S	$C_{37}H_{66}N_7O_{17}P_3S$	No	1005.3448	1005.3450	0.2	0.46
16:1 Conenzyme A	16:1-CoA	D	$C_{37}H_{64}N_7O_{17}P_3S$	No	1003.3384	1003.3280	-10.3	na
18:0 Conenzyme A	18:0-CoA	D	$C_{39}H_{70}N_7O_{17}P_3S$	No	1033.3761	1033.3688	-7.0	na
18:1 Conenzyme A	18:1-CoA	D	$C_{39}H_{68}N_7O_{17}P_3S$	No	1031.3605	1031.3590	-1.4	na
**Sugar Nucleotide Donors**								
GDP- mannose	GDP-M	S	$C_{16}H_{25}N_5O_{16}P_2$	No	605.0771	605.0780	1.5	4.2
UDP-d-galacturonate	UDP-GA	D	$C_{15}H_{22}N_2O_{18}P_2$	No	580.0343	580.0334	-1.5	na
GDP-fucose	GDP-F	D	$C_{16}H_{25}N_5O_{15}P_2$	No	589.0822	589.0827	0.9	na
UDP-GlcNAc+GalNAc	UDP-GlcNAc + GalNAc	D	$C_{17}H_{27}N_3O_{17}P_2$	No	607.0816	607.0814	-0.3	na
UDP-xylose	UDP-X	D	$C_{14}H_{22}N_2O_{16}P_2$	No	536.0444	536.0408	-6.7	na
UDP-glucose + UDP-galactose	UPD-Glc + UDP-Gal	S	$C_{15}H_{24}N_2O_{17}P_2$	No	566.0550	566.0528	-3.9	7.3
**Miscellaneous**								
aminoimidazole carboxamide ribonucleotide	ZMP	S	$C_9H_{15}N_4O_8P$	No	338.0627	338.0614	-3.8	4
glycineamideribotide	GAR	D	$C_7H_{15}N_2O_8P_1$	No	286.0566	286.0577	3.9	na
phosphocreatine	PCRE	S	$C_4H_{10}N_3O_5P$	No	211.0358	211.0351	-3.2	120
creatine	CRE	S	$C_4H_9N_3O_2$	No	131.0695	131.0703	6.3	8.4
phosphate	PO4	S	H_4O_4P	No	97.9769	97.9772	3.4	383
pantothenic acid	PAN	S	$C_9H_{17}NO_5$	No	219.1107	219.1120	6.0	0.035
2-o-(6-phospho-alpha-mannosyl)-d-glycerate	PMG	D	$C_9H_{17}O_{12}P_1$	No	348.0458	348.0432	-7.4	na
cytidine diphosphate- ethanolamine	CDP-EA	D	$C_{11}H_{20}N_4O_{11}P_2$	No	446.0604	446.0594	-2.2	na
beta-aspartylglycine	DG	D	$C_6H_{10}N_2O_5$	No	190.0590	190.0594	2.2	na
citicoline	CC	D	$C_{14}H_{26}N_4O_{11}P_2$	No	488.1073	488.1060	-2.6	na
farnesyl pyrophosphate	FPP	D	$C_{15}H_{28}O_7P_2$	No	382.1310	382.1296	-3.6	na
L-beta-aspartyl-L-alanine or 5-L-Glutamylglycine	DA or EG	D	$C_7H_{12}N_2O_5$	No	204.0746	204.0748	1.1	na
glycerylphosphorylethanolamine	GPEA	D	$C_5H_{14}NO_6P$	No	215.0559	215.0551	-3.6	na
Hexol phosphates	MP	D	$C_6H_{15}O_9P$	No	262.0454	262.0443	-4.1	na
HMG-CoA	HMG-CoA	S	$C_{27}H_{44}N_7O_{20}P_3S$	No	911.15744	911.1548	-2.9	na
**Free Fatty Acids**								
16:0	16:0	S	$C_{16}H_{32}O_2$	No	256.2402	256.2396	-2.3	na
18:0	18:0	S	$C_{18}H_{36}O_2$	No	284.2715	284.2712	-1.0	na
18:1	18:1	S	$C_{18}H_{34}O_2$	No	282.2559	282.2554	-1.7	na
18:2	18:2	D	$C_{18}H_{32}O_2$	No	280.2402	280.2398	-1.4	na
20:0	20:0	S	$C_{20}H_{40}O_2$	No	312.3028	312.3011	-5.4	na
20:1	20:1	D	$C_{20}H_{38}O_2$	No	310.2872	310.2872	0.1	na
20:2	20:2	D	$C_{20}H_{36}O_2$	No	308.2715	308.2711	-1.2	na
22:0	22:0	S	$C_{22}H_{44}O_2$	No	340.3341	340.3335	-1.7	na
22:1	22:1	D	$C_{22}H_{42}O_2$	No	338.3185	338.3193	2.4	na
22:2	22:2	D	$C_{22}H_{40}O_2$	No	336.3028	336.3031	1.0	na
